# Disruption of actin dynamics induces autophagy of the eukaryotic chaperonin TRiC/CCT

**DOI:** 10.1038/s41420-022-00828-6

**Published:** 2022-01-25

**Authors:** Yuki Date, Akira Matsuura, Eisuke Itakura

**Affiliations:** 1 Department of Biology, Graduate School of Science and Engineering, Chiba University, Inage-ku, Chiba, 263-8522 Japan; 2 Department of Biology, Graduate School of Science, Chiba University, Inage-ku, Chiba, 263-8522 Japan

**Keywords:** Protein quality control, Macroautophagy

## Abstract

Autophagy plays important role in the intracellular protein quality control system by degrading abnormal organelles and proteins, including large protein complexes such as ribosomes. The eukaryotic chaperonin tailless complex polypeptide 1 (TCP1) ring complex (TRiC), also called chaperonin-containing TCP1 (CCT), is a 1-MDa hetero-oligomer complex comprising 16 subunits that facilitates the folding of ~10% of the cellular proteome that contains actin. However, the quality control mechanism of TRiC remains unclear. To monitor the autophagic degradation of TRiC, we generated TCP1α-RFP-GFP knock-in HeLa cells using a CRISPR/Cas9-knock-in system with an RFP-GFP donor vector. We analyzed the autophagic degradation of TRiC under several stress conditions and found that treatment with actin (de)polymerization inhibitors increased the lysosomal degradation of TRiC, which was localized in lysosomes and suppressed by deficiency of autophagy-related genes. Furthermore, we found that treatment with actin (de)polymerization inhibitors increased the association between TRiC and unfolded actin, suggesting that TRiC was inactivated. Moreover, unfolded actin mutants were degraded by autophagy. Taken together, our results indicate that autophagy eliminates inactivated TRiC, serving as a quality control system.

## Introduction

Autophagy, an important quality control system of proteins in a cell, is a housekeeping cleaning process that has been conserved in organisms from yeast to mammals. An isolation membrane (also known as a phagophore) is formed and expands to envelop substrates in the cytosol. Then, the isolation membrane closes to form a double-membrane structure called an autophagosome [1–4]. Autophagosomes fuse with lysosomes, leading to degradation of the substrates by lysosomal enzymes [5]. In mammals, nutrient starvation primarily induces bulk autophagy, which randomly engulfs a portion of the cytoplasm to generate amino acids via lysosomal degradation. On the other hand, autophagy is induced by diverse conditions and specifically targets substrates for degradation to maintain cell homeostasis associated with various diseases.

Autophagy selectively targets damaged cytoplasmic proteins and organelles for lysosomal degradation. In mammals, mitophagy, the mechanism by which dysfunctional mitochondria are degraded by autophagy, has been well characterized. Depolarized mitochondria stabilize the PINK1 protein on mitochondria and recruit the Parkin E3 ligase, which ubiquitinates outer mitochondrial membrane proteins. Then, these ubiquitinated mitochondria recognized by autophagy adaptor proteins are enclosed by an autophagosome [6, 7]. Hypoxia and reactive oxygen species also induce ubiquitin-independent mitophagy via outer mitochondrial membrane-resident mitophagy receptors such as BNIP3/NIX, FUNDC1, Bcl2L13, and FKBP8 [8]. These mitophagy receptors inserted on mitochondria bind with LC3 to dock mitochondria to the isolation membrane. Autophagy degrades various other substrates under stress conditions. ERphagy, which degrades the endoplasmic reticulum (ER), is induced by starvation or ER stress [9, 10]. Endosome rupture triggers xenophagy and lysophagy, which degrade invading bacteria and lysosomes, respectively [11]. A recent study revealed that large protein complexes, including ribosomes, ferritin, and proteasomes, are degraded and subjected to the quality control system by autophagy under various stress conditions. Ribophagy is induced by translational inhibition by arsenite, initiating ribosomal quality control [12, 13]. Iron deficiency leads to the activation of ferritinophagy, in which ferritin is degraded to release iron [14]. Proteaphagy is induced by proteotoxic stress [15]. Thus, autophagy preferentially eliminates damaged large structures on the basis of a specific type of stress. Therefore, we hypothesized that autophagy plays a role in quality control for other large protein complex(es) under stress.

The eukaryotic chaperonin tailless complex polypeptide 1 (TCP1) ring complex (also called chaperonin-containing, TCP1) (TRiC/CCT) is an essential eukaryotic chaperonin that hydrolyzes ATP to facilitate the folding of nascent polypeptide chains and denatured proteins. TRiC is a 16-subunit complex of almost 1 MDa that consists of two back-to-back stacked rings that each contains eight different subunits of approximately 60 kDa [16]. The complex forms a barrel-like and cavity structure for the ATP-driven chaperonin-mediated protein-folding cycle. The dynamic conformational change induced by ATP hydrolysis may be closely associated with the productive folding of the substrate. Since TRiC facilitates the folding of approximately 10% of cytosolic proteins, including the cytoskeleton proteins actin and tubulin, which are representative substrates of TRiC [17–19], TRiC is essential for cell viability, and plays multiple roles in cell migration, cell division, and cell growth [20, 21]. While the physiological functions and folding mechanism of TRiC are well understood, the quality control mechanism of TRiC remains unclear.

To analyze the quality control system of TRiC, we established TCP1α-RFP-GFP (TCP1α-RG) knock-in HeLa cells using a CRISPR/Cas9-knock-in system that enabled quantification of lysosome-dependent degradation of TRiC through comparisons of the RFP/GFP ratio. We confirmed that almost all TCP1α-RG were incorporated into endogenous TRiC in the knock-in cells, treated the knock-in cells with various drugs that induce stress, and analyzed the degree of protein lysosomal degradation using a flow cytometer. We found that cytochalasin D, an inhibitor of actin polymerization, induced the lysosomal degradation of TRiC. Moreover, cytochalasin D-induced degradation of TRiC was dependent on autophagy-related genes, and cytochalasin D treatment increased the association between TRiC and nonnative actin, resulting in the inactivation of TRiC. These data suggest that autophagy degrades the TRiC complex inactivated by a disorder of actin.

## Results

### Generation of knock-in cells endogenously expressing TCP1α-RFP-GFP

**Fig. 1:**
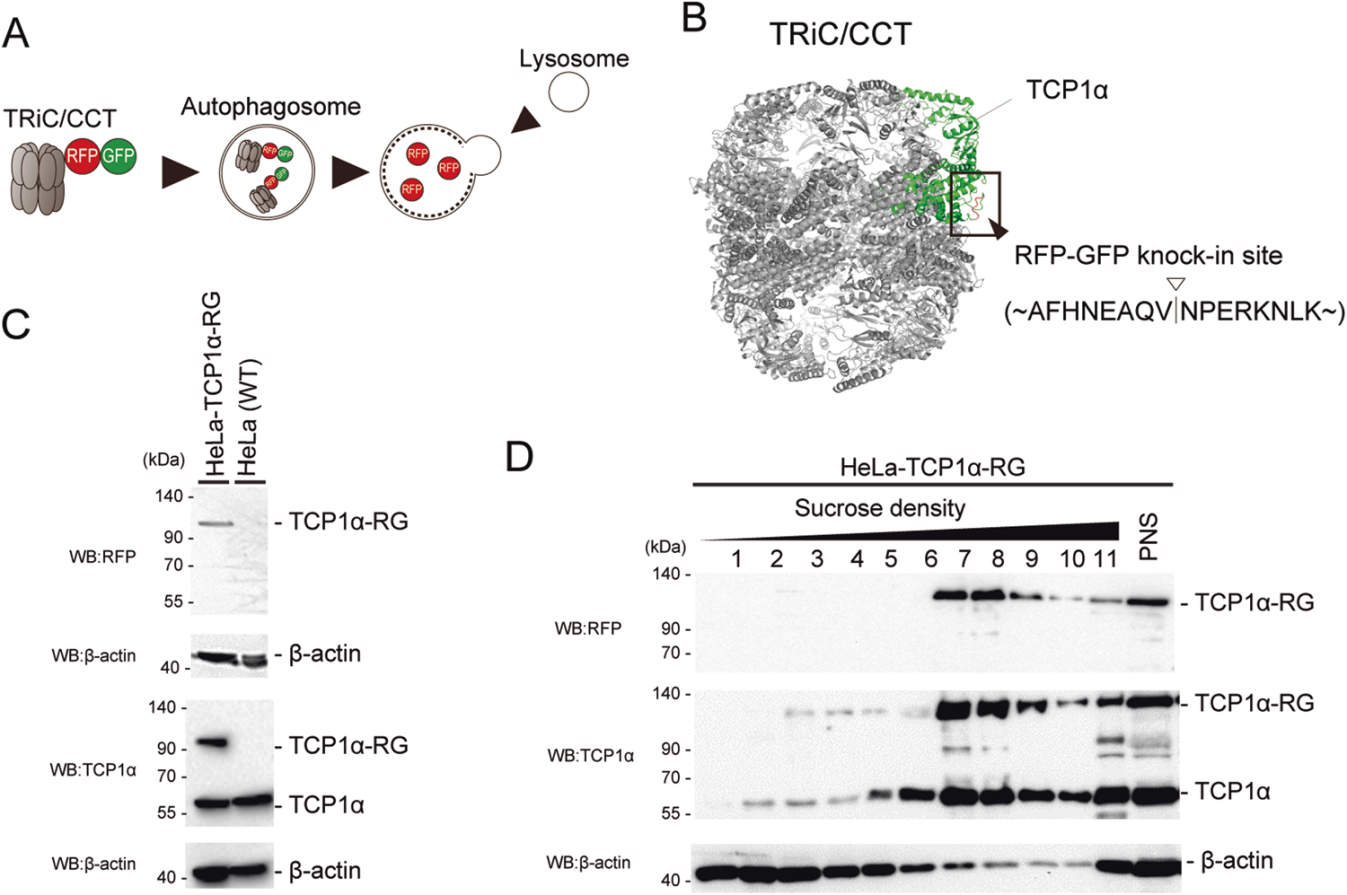
Generation of HeLa TCP1α-RFP-GFP (RG) knock-in cells. **A** Schematic diagram of the autophagic degradation of TRiC that is fused with RFP-GFP (RG). In lysosomes, TRiC and GFP are degraded. In contrast, free RFPs are accumulated in lysosomes. **B** View of the position of the RFP-GFP fusion to the TRiC complex. The TCP1α subunit is shown in green, and the knocked-in loop site is shown in red. **C** Immunoblot of HeLa cells endogenously expressing TCP1α-RFP-GFP (HeLa TCP1α-RG) generated by the CRISPR/Cas9 knock-in system. Cells were lysed in lysis buffer and analyzed by immunoblotting using antibodies against RFP, TCP1α, and β‐actin. **D** Knock-in TCP1α-RG forms TRiC. HeLa TCP1α-RG cells were homogenized, and the resultant postnuclear supernatant (PNS) was fractionated by sucrose density gradients and analyzed by immunoblotting using antibodies against RFP, TCP1α, and β‐actin.

To measure lysosomal degradation of TRiC, we fused the RFP-GFP tandem tag to TCP1α, a subunit of TRiC. After transport of RFP-GFP into mammalian lysosomes, GFP is pH-sensitive and degraded by lysosomal proteases. In contrast, RFP is resistant to acidic conditions and lysosomal proteases, resulting in its accumulation in lysosomes [22]. These characteristics of RFP and GFP enabled us to sensitively evaluate the autophagic degradation of TRiC by measuring the fluorescence ratio of RFP/GFP in a cell using flow cytometry and immunoblotting to detect cleaved RFP (Fig. 1A). Fusion of the RFP-GFP tandem tag to the C- or N-terminus of a CCT subunit of TRiC blocks TRiC complex formation and inhibits substrate folding [23]. To fuse RFP-GFP outside of the TRiC structure, RFP-GFP was inserted into the central loop region (amino acids between 476 and 477) of TCP1α, which did not interfere with complex formation, as previously reported [23] (Fig. 1B).

We generated HeLa cells stably expressing exogenous TCP1α-RFP-GFP (TCP1α-RG). However, most of the TCP1α-RG were not incorporated into the endogenous TRiC (Fig. S1A). Therefore, we established a knock-in cell line (HeLa TCP1α-RG cells) in which endogenous TCP1α was tagged with RFP-GFP by CRISPR/Cas9. A targeting RFP-GFP vector with 700 bp homologous arms and a Cas9 vector containing gRNA, which targets exon 11 of TCP1α, were transfected into HeLa cells. The RFP- and GFP-positive cells were single sorted and expanded. Immunoblot analysis revealed the expected 120 kDa band by anti-RFP and anti-TCP1α antibodies (Fig. 1C). Notably, endogenous TCP1α was still observed, suggesting heterogeneous knock-in. A sucrose density gradient analysis of cell lysates showed that TCP1α-RG migrated exclusively to TRiC fractions (Fig. 1D). In addition, MTT assay showed that the proliferation rate of HeLa TCP1α-RG cells is almost identical to wild-type cells (Fig. S1B). These results indicate that the RFP-GFP construct was incorporated into the TRiC genomic region in the chromosome(s) of HeLa cells.

### Actin (de)polymerization inhibitors induce degradation of TRiC via lysosomes

TRiC folds approximately 10% of cytosolic protein using the hydrolytic energy of ATP, and the main substrates are actin and tubulin. ATP induces dynamic structural changes in TRiC, causing it to switch between open and closed states [24]. We speculated that the dynamic nature of TRiC collapses under stress conditions, leading to the removal of stressed TRiC by autophagy.

**Fig. 2:**
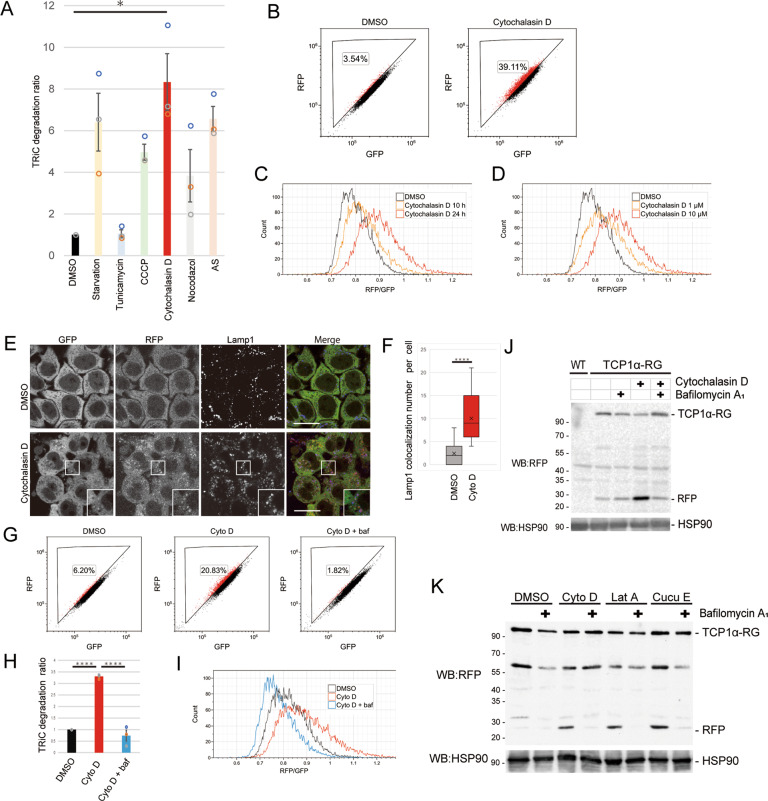
Cytochalasin D treatment induces lysosomal degradation of TRiC. **A**, **B** Analysis of lysosomal degradation of TRiC by flow cytometry. HeLa TCP1α-RG cells were treated with tunicamycin, CCCP, cytochalasin D, nocodazole, or arsenic for 24 h and analyzed by flow cytometry. Dot plots show the lysosomal TRiC gate-positive cell population (%). The bar graph shows the TRiC degradation ratio: the compound-treated lysosomal TRiC gate-positive cell population (%)/DMSO-treated lysosomal TRiC gate-positive cell population (%) (*n* = 3). The data are presented as the means ± SEM. *P < 0.05, t-test. **C**, **D** Time- and concentration-dependent lysosomal TRiC degradation by cytochalasin D. HeLa TCP1α-RG cells were treated with DMSO or cytochalasin D (C: 10 µM for 10 or 24 h, C) (D: 1 or 10 μM for 24 h) and analyzed by flow cytometry. The histogram shows the number of cells on the vertical axis and the RFP/GFP ratio on the horizontal axis. **E** TRiC-RG is transported to lysosomes. HeLa TCP1α-RG cells were treated with DMSO or cytochalasin D for 24 h, fixed and stained with anti-lamp1 antibody, and then analyzed by confocal microscopy. Scale bar, 20 µm. **F** The box plot shows the RFP/Lamp1 colocalization in HeLa TCP1α-RG cells treated with DMSO or cytochalasin D (Cyto D) for 24 h (*n* = 30 cells). **** *P* < 0.0001, t-test. **G**–**I** Lysosomal TRiC degradation is suppressed by bafilomycin A_1_ treatment. HeLa TCP1α-RG cells were treated with DMSO or cytochalasin D with or without bafilomycin A_1_ (baf) for 24 h and analyzed by flow cytometry. Dot plots show the lysosomal TRiC gate-positive cell population (%). The bar graph shows the TRiC degradation ratio of H (*n* = 3). The data are presented as the means ± SEM. *P < 0.05, ANOVA Tukey’s test. **J** Analysis of lysosomal TRiC degradation by TCP1α-RG cleavage assay. HeLa TCP1α-RG cells were treated with DMSO or cytochalasin D, with or without bafilomycin A_1_ (baf) for 24 h, and analyzed by immunoblotting using antibodies against RFP and HSP90. **K** Suppression of actin dynamics causes lysosomal TRiC degradation. HeLa TCP1α-RFP-GFP cells were treated with DMSO, cytochalasin D (Cyto D), latrunculin A (Lat A) or cucurbitacin E (Cucu E) with or without bafilomycin A_1_ (baf) for 24 h and analyzed by immunoblotting using antibodies against RFP and HSP90.

We first validated the autophagic degradation of TCP1α-RG by cell nutrient starvation and observed a decrease in GFP but not in RFP fluorescence, resulting in a shift of the cell population into the lysosomal TRiC gate during fluorescence flow cytometry (Fig. S2A). To induce TRiC stress, HeLa TCP1α-RG cells were cultured with various cell stress inducers (tunicamycin (an ER stress inducer), CCCP (a mitochondrial uncoupler), cytochalasin D (an inhibitor of actin polymerization), nocodazole (an inhibitor of microtubule polymerization) and arsenic (an inhibitor of translation)) for 24 h and then analyzed by flow cytometry. The RFP and GFP intensities in each cell were measured, and the lysosomal degradation ratio was calculated as follows: the compound-treated lysosomal TRiC-positive cell population (%)/DMSO-treated lysosomal TRiC-positive cell population (%). The results revealed that cytochalasin D treatment resulted in the greatest increase in the lysosomal degradation ratio (Figs. 2A, B, and S2B). The increase in the RFP/GFP ratio with cytochalasin D treatment was time- and concentration-dependent (Fig. 2C, D). Cytochalasin D treatment drastically increased the accumulation of RFP dots, which colocalized with lamp1, a lysosomal membrane protein (Fig. 2E, F). In addition, treatment with a lysosomal acidification inhibitor, bafilomycin A_1_, inhibited the increase in the lysosomal degradation ratio induced by cytochalasin D (Fig. 2G–I). Taking advantage of protease resistant properties of RFP in mammalian lysosomes, we then confirmed lysosomal degradation with a TCP1α-RG cleavage assay used to evaluate the accumulation of cleaved RFP (~25 kDa), reflecting the degradation of TCP1α, GFP, or linker regions by lysosomal hydrolases. Cleaved RFP was increased by treatment with cytochalasin D and was suppressed by that with bafilomycin A_1_ treatment (Fig. 2J). Other actin depolymerizers, namely, latrunculin A, an inhibitor of actin polymerization, and cucurbitacin E, an inhibitor of actin depolymerization, also induced lysosomal degradation of TCP1α-RG (Fig. 2K). These results suggest that suppression of actin dynamics induced TRiC degradation by lysosomes.

### Autophagy degrades TRiC under stress conditions that inactivate actin function

**Fig. 3:**
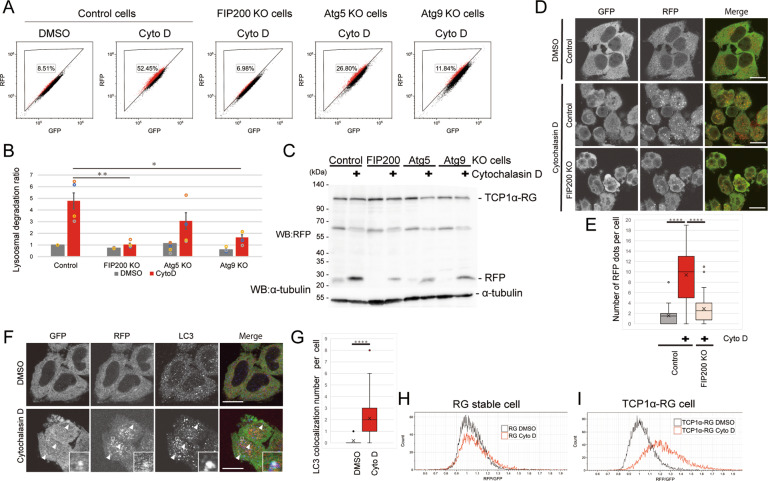
Lysosomal TRiC degradation is dependent on autophagy. **A**–**C** Autophagy KO inhibits lysosomal degradation of TRiC-RG. Control (nontargeting gRNA for the human genome), FIP200-, Atg5-, or Atg9-knockout (KO) HeLa TCP1α-RG cells were treated with DMSO or cytochalasin D (Cyto D) for 24 h and analyzed by flow cytometry. Dot plots show the lysosomal TRiC gate-positive cell population (%) (**A**). The bar graph shows the TRiC degradation ratio of A (*n* = 4). The data are presented as the means ± SEM. *P < 0.05, **P < 0.01, ANOVA and Dunnett’s test (**B**). Cells were treated as in A and analyzed by immunoblotting using antibodies against RFP and α-tubulin (**C**). **D** The number of RFP puncta were decreased in autophagy-deficient cells. Control and FIP200 KO HeLa TCP1α-RG cells were treated with DMSO or cytochalasin D (Cyto D) for 24 h, fixed, and analyzed by confocal microscopy. Scale bar, 20 µm. **E** The box plot shows RFP puncta per cell (*n* = 30 cells). ****P < 0.0001, ANOVA and Dunnett’s test. **F** TCP1α-RG colocalizes with autophagosomes. HeLa TCP1α-RFP-GFP cells were treated with DMSO or cytochalasin D for 24 h, fixed and stained with anti-LC3, and then analyzed by confocal microscopy. Scale bar = 20 µm. **G** The box plot shows the RFP/LC3 colocalization in HeLa TCP1α-RFP-GFP cells treated with DMSO or cytochalasin D (Cyto D) for 24 h (*n* = 30 cells). **** *P* < 0.0001, t-test. **H**, **I** HeLa RF*P*-GFP cells (RG) and HeLa TCP1α-RFP-GFP (TCP1α-RG) cells were treated with DMSO or cytochalasin D for 24 h and analyzed by flow cytometry. Histogram shows RFP/GFP ratio per cell.

To investigate whether lysosome-dependent degradation of TRiC is dependent on autophagy, we generated HeLa knock-in TCP1α-RG cells that exhibit defective autophagy (FIP200-, Atg9- or Atg5-knockout (KO) cells (Fig. S3), which are required for early or late stages of autophagosome formation, respectively) by CRISPR/Cas9 [25–27]. A flow cytometry analysis showed that the increase in the cell population counted in the lysosomal TRiC gate that had been induced by cytochalasin D was significantly reduced in the FIP200- and Atg9- cells compared to the control cells (Fig. 3A, B). On the other hand, Atg5 KO cells showed only a marginal difference in the analysis. Through a TCP1α-RG cleavage assay, the accumulation of cleaved RFP was found to be markedly reduced in the KO cells (Fig. 3C). The cleaved RFP was still observed in the KO cells, because the KO cells are not monoclonal KO cell lines, in which gene mutations are induced by expression of gRNA and Cas9 via lentivirus (Note that the polyclonal KO cells were used as a KO cell line, and fifty to ninety percent of cells were defect in autophagy). We confirmed that the increase in RFP puncta of TCP1α-RG induced by cytochalasin D treatment was suppressed in FIP200 KO cells (Fig. 3D, E), indicating that autophagosomes engulfed TRiC after cytochalasin D treatment. Indeed, an immunostaining analysis revealed that GFP and RFP puncta in TCP1α-RG cells colocalized with LC3 (Fig. 3F, G). These results suggest that autophagy is required for lysosomal degradation of TRiC under actin stress conditions. There are two possibilities for this autophagy-dependent degradation: cytochalasin D induced bulk autophagy that led to nonselectively engulfed cytosolic proteins, including TCP1α-RG, or cytochalasin D preferentially induced autophagic degradation of TCP1α-RG. To confirm the latter possibility, we compared the lysosomal degradation ratio between HeLa TCP1α-RG cells and HeLa cells stably expressing RFP-GFP alone by performing flow cytometry. While cytochalasin D drastically increased the lysosomal degradation ratio in the HeLa TCP1α-RG cells, the ratio was not significantly increased in the HeLa RFP-GFP cells (Fig. 3H, I). These results imply that TRiC was preferentially degraded by autophagy.

### TRiC is inactivated by cytochalasin D treatment

**Fig. 4:**
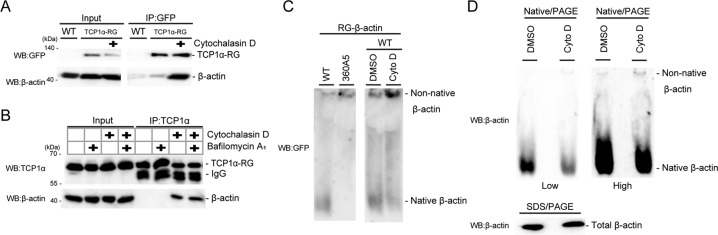
TRiC is inactivated by cytochalasin D treatment. **A** The interaction between TRiC-RG and β-actin was enhanced under treatment with cytochalasin D. HeLa TCP1α-RG cells were treated with DMSO or cytochalasin D for 24 h, lysed in lysis buffer and subjected to immunoprecipitation using GFP-Trap beads. **B** Interaction between endogenous TRiC and β-actin was enhanced under treatment with cytochalasin D. HeLa WT cells were treated with DMSO or cytochalasin D (Cyto D) with or without bafilomycin A_1_ (baf) for 24 h, lysed by lysis buffer and subjected to immunoprecipitation using anti-TCP1α antibody. **C** Cytochalasin D treatment increases nonnative exogenous β-actin. Left panel: HeLa cells stably expressing RG-β-actin WT or RG-β-actin 360A5 were analyzed by native PAGE and immunoblotting using antibodies against GFP. Right panel: HeLa cells stably expressing RG-β-actin WT were treated with DMSO or cytochalasin D, lysed by lysis buffer, and analyzed by native/PAGE and immunoblotting using antibodies against GFP. **D** Cytochalasin D treatment increases nonnative endogenous β-actin. HeLa cells were treated with DMSO or cytochalasin D, lysed in lysis buffer, and analyzed by native PAGE and immunoblotting using antibodies against β-actin. Bottom panel: samples boiled in SDS buffer were analyzed by immunoblotting using antibodies against β-actin.

Considering our results, we wondered how cytochalasin D impacts TRiC. We hypothesized that disturbance of actin dynamics by actin (de)polymerization retards TRiC actin-folding activity. To investigate the interaction between TRiC and actin, we performed coimmunoprecipitation assays using anti-GFP and anti-TCP1α antibodies. While TCP1α-RG and endogenous TCP1α did not coprecipitate with β-actin under normal conditions, after cytochalasin D treatment the interaction between TCP1α and β-actin was apparent (Fig. 4A, B). An actin depolymerization inhibitor (cucurbitacin E) also increased the association of TRiC with β-actin, whereas the polymerization inhibitor latrunculin A did not affect this association (Fig. S4A). Because latrunculin A directly binds to monomeric β-actin, it might mask the interaction region between β-actin and TRiC. We speculated that the increase in the interaction of TRiC-actin is indicative of TRiC occupation with unfolded actin. To confirm this hypothesis, we used the β-actin 360A5 mutant, which causes folding defects, to which TRiC shows high affinity in vitro [28, 29], and performed an actin folding assay with native/PAGE. We prepared cells stably expressing RFP-GFP(RG)-β-actin wild-type (WT) or RG-β-actin 360A5, and each lysate was separated by native/PAGE and analyzed by immunoblotting using anti-GFP and anti-β-actin antibodies. The RG-β-actin WT migrated faster in the gel, representing native actin (Fig. 4C (left)). In contrast, RG-β-actin 360A5 migrated much slower than RG-β-actin WT, which was indicative of nonnative actin (Fig. 4C (left)). We next performed the same experiment using cell lysates treated with DMSO or cytochalasin D. Interestingly, cytochalasin D treatment reduced native actin and strikingly increased nonnative actin (Fig. 4C (right)). This finding was verified by endogenous β-actin (Fig. 4D). Other actin (de)polymerization inhibitors, namely, cucurbitacin E and latrunculin A, also reduced native actin (Fig. S4B). These results suggest that actin (de)polymerization inhibitor treatment led to the accumulation of TRiC-actin in the intermediate state.

### Autophagy degrades the TRiC-actin complex

**Fig. 5:**
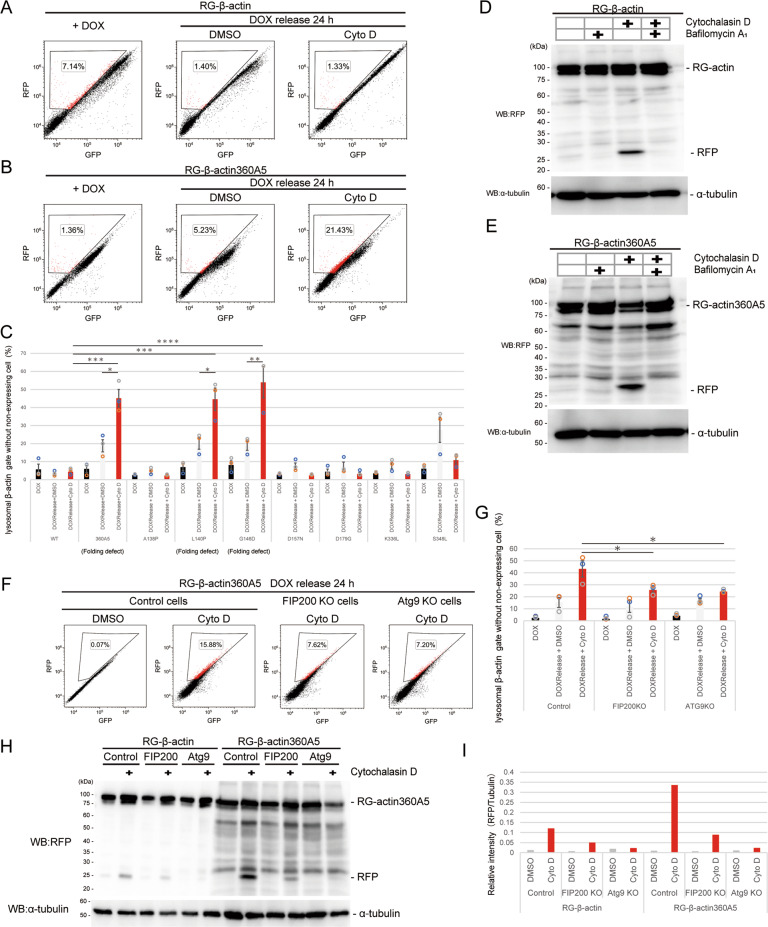
Autophagy degrades folding-retarded β-actin. **A**–**C** Lysosomal degradation of folding-retarded β-actin mutants. HeLa tetracycline‐on (Tet‐on) cells expressing WT RFP-GFP(RG)-β-actin (**A**) or RG-β-actin folding mutants (**B**, 360A5) (**C**, other mutants) were cultured in the presence of doxycycline (Dox). After removal of Dox, the cells were treated with DMSO or cytochalasin D (Cyto D) for 24 h and analyzed by flow cytometry. Dot plots show the lysosomal β-actin gate-positive cell population (%). The graph shows the lysosomal β-actin gate (%) excluding nonexpressing cells (*n* = 3). The data are presented as the means ± SEM. * *P* < 0.05, ** *P* < 0.01, *** *P* < 0.001, **** *P* < 0.0001, ANOVA and Turkey’s test. **D**, **E** HeLa Tet‐on cells expressing RFP-GFP(RG)-β-actin or RG-β-actin 360A5 were cultured in the presence of doxycycline (Dox) and treated with DMSO or cytochalasin D (Cyto D) with or without bafilomycin A_1_ for 24 h and then analyzed by immunoblotting using antibodies against RFP and α-tubulin. **F** Control (nontargeting gRNA of the human genome), FIP200- or Atg9-KO HeLa Tet‐on cells expressing RG-β-actin 360A5 were treated with DMSO or cytochalasin D (Cyto D) for 24 h and analyzed by flow cytometry. Dot plots show the lysosomal β-actin-positive-gate cell population. **G** The graph shows the lysosomal β-actin gate (%) excluding nonexpressing cells (*n* = 3). The data are presented as the means ± SEM. *P < 0.05, ANOVA and Dunnett’s test. **H** Control, FIP200- or Atg9-KO HeLa Tet‐on cells expressing RFP-GFP(RG)-β-actin or RG-β-actin 360A5 were cultured in the presence of doxycycline (Dox), treated with DMSO or cytochalasin D (Cyto D) for 24 h, and then analyzed by immunoblotting using antibodies against RFP and α-tubulin. **I** Relative level of cleaved RFP. The bar graph shows RFP/tubulin ratio of intensity.

If β-actin-bound TRiC were degraded by autophagy upon cytochalasin D treatment, cytochalasin D would also induce autophagic degradation of β-actin. To detect autophagic degradation of β-actin, HeLa cells expressing RG-β-actin WT or RG-β-actin 360A5 under a tetracycline-inducible promoter (Tet-on) were tested. After inducing the expression of RG-actin and RG-β-actin 360A5 for 24 h by adding doxycycline (DOX), DOX was removed from the medium to stop the new synthesis of RG-β-actin, and then the cells were cultured with or without cytochalasin D for 24 h. In the cells expressing RG-β-actin WT, cytochalasin D treatment did not elevate the population of cells in the lysosomal actin gate (Fig. 5A). This outcome might have been due to the expression of exogenous RG-β-actin WT being lower than that of endogenous β-actin (Fig. S5A). In contrast, in the cells expressing RG-actin 360A5, an increase in the population of cells in the lysosomal actin gate was remarkably increased after cytochalasin D treatment (Fig. 5B, C). Confocal microscopy showed that the cells expressing RG-β-actin 360A5, but not those expressing β-actin WT, exhibited increased RFP puncta upon treatment with cytochalasin D (Fig. S6A). The lysosomal degradation of RG-actin was verified by an RG-actin cleavage assay, which showed an increase in cleaved RFP upon cytochalasin D treatment not only in RG-β-actin 360A5 but also in RG-β-actin WT cells (Fig. 5D, E). In addition, the accumulation of cleaved RFP was abrogated by bafilomycin A_1_ treatment (Fig. 5D, E) and led to defective autophagy (FIP200- and Atg9-KO) cells showed reduced degradation of both RG-β-actin WT and RG-β-actin 360A5 (Fig. 5F–I), suggesting that cytochalasin D induced autophagy that degraded the TRiC-actin complex. Moreover, we performed a similar experiment using various actin mutants involved in myopathy [30]. The results showed that two actin unfolding mutants (L140P and G146D) were significantly degraded by autophagy compared with other dysfunctional mutants (β-actin A138P, D157N, D179G, K336L, and S348L) (Figs. 5C, S5B, and S6B). Since β-actin 360A5, L140P and G146D were unfolded mutants and trapped in TRiC in vitro [28], unfolded actin may occupy a cavity in TRiC for a long time, resulting in functional inhibition of the complex. These results suggest that TRiC with unfolded actin is targeted to autophagy.

## Discussion

In this study, we sought to determine whether autophagy degrades a 1-MDa protein complexes, TRiC [16]. We fused RFP-GFP to endogenous TCP1α, one of the components of TRiC, using CRISPR/Cas9 and generated HeLa TCP1α-RG cells in which the lysosomal degradation of TRiC could be quantified by comparing the fluorescence ratio (Fig. 1). We identified that actin (de)polymerization inhibitors induced degradation of TRiC by autophagy (Figs. 2 and 3). Furthermore, we found that actin (de)polymerization inhibitors increased the interaction of TRiC with actin and reduced its folding activity (Fig. 4). These results suggest that autophagy acts as a quality control system for inactivated TRiC.

Our autophagy assay using TCP1α-RG revealed autophagic degradation of TRiC by autophagy not only upon treatment with actin (de)polymerization inhibitors but also under basal conditions (Fig. 3C). This finding is consistent with a previous report showing that a global proteomic analysis of autophagy-deficient cells exhibits basal turnover of TRiC through autophagy [31, 32]. Autophagy-deficient cells might accumulate inactivated TRiC; however, performing an actin folding assay, we did not detect reduced TRiC activity in autophagy KO cells (data not shown). This result may have been due to the dilution effect in which the level of aberrant TRiC is diluted by cell division.

What is the mechanism by which actin (de)polymerization inhibitors lead degradation of TRiC? Actin (de)polymerization inhibitors originally target actin but not TRiC, indicating that the inhibitors do not directly inhibit TRiC. One possible explanation of (de)polymerization inhibitor action is that actin inhibitors disturb actin dynamics, which might lead to retardation of actin maturation. As a result, TRiC may exhibit prolonged interaction with immature actin, and this complex may be recognized as abnormal TRiC. Indeed, we showed that cytochalasin D treatment increased TRiC interacting with unfolded actin (Fig. 4), and efficient autophagic degradation of TRiC was observed 24 h after treatment with cytochalasin D (Figs. 2 and 3). In addition, in a previous study, in vitro actin folding using purified TRiC indicated that actin folding by TRiC could be realized within 30 min [33]. Therefore, the degradation of TRiC can be explained by considering a timer model that had been previously used for ER-associated degradation (ERAD) [34]. ERAD distinguishes bona fide folding intermediates and misfolded proteins by time, specifically, the length of time that a glycoprotein spends in the ER. Indeed, activation of ERAD requires more than 30–90 min [35]. Accordingly, TRiC that maintains a complex with actin for longer than required to fold it may trigger autophagic degradation.

Another possibility is that cytochalasin D might induce the inactivation of TRiC via phosducin-like protein (PhLP), which is a regulator of TRiC activity [36–39]. For example, phosphorylated PhLP promotes Gβ folding, whereas unphosphorylated PhLP binds to TRiC with Gβ, leading to inactivation of TRiC in the trimeric state [36, 39]. Indeed, PhLP with TRiC-actin inhibits actin folding in the trimeric state as well [37]. According to our data, native β-actin was significantly reduced by cytochalasin D (Fig. 4C, D), implying that cytochalasin D treatment promotes PhLP binding to TRiC. The PhLP family is regulated by phosphorylation of casein kinase 2 (CK2) and G-protein-coupled receptor kinase 2 (GRK2) [36, 39, 40]. CK2 is involved in proliferation and survival [41, 42] and negatively regulates actin polymerization via phosphorylation of CRN2 [43]. Although the roles played by upstream factors of CK2 in actin regulation are not well understood, CK2 is activated by ERK [44]. GRK2 is also phosphorylated by ERK [45, 46]. Interestingly, the activity of ERK is inhibited by actin (de)polymerization inhibitors [47, 48]. These reports might support the supposition that the disturbance of actin filaments results in inactivation of CK2 and GRK2. Therefore, it is assumed that inhibition of the ERK signaling cascade acts as negative feedback to halt actin folding via dephosphorylation of PhLP in response to actin stress and thus regulates actin homeostasis.

Cytochalasin D might induce selective autophagy of TRiC because the level of autophagic degradation of TCP1α-RG, but not RG, was significantly increased by treatment with cytochalasin D (Fig. 3H, I). Several autophagy adaptor proteins are known to recruit autophagic substrates to the autophagosomal membrane [49]. To identify an autophagic adaptor protein in autophagic degradation of TRiC, we tried various experiments using known autophagic adaptors and found that p62 was coprecipitated with endogenous TCP1α in the presence of a (de)polymerization inhibitor with bafilomycin A_1_ (data not shown). In addition, p62 colocalized with TCP1α-RG after treatment with cytochalasin D (data not shown). However, p62-KO cells did not exhibit any defects in the degradation of TCP1α-RG. Although we tested cells in which known autophagic adaptors were knocked out, including NBR1, NDP52, optineurin, TAX1BP1, and TOLLIP, these KO cells showed normal degradation of TCP1α-RG upon treatment with cytochalasin D (Fig. S7). These autophagic adaptor proteins basically target ubiquitinated substrates for recognition. However, ubiquitination of TCP1α under treatment with cytochalasin D was not detected in our experiments. Additionally, cells with ubiquitin-independent autophagy adaptor proteins knocked out, including TRIM5, NUFIP, TOLLIP or NCOA4, did not show impaired degradation of TRiC. Therefore, autophagosomes recognize TRiC via unidentified autophagic adaptor(s). Further study is necessary to identify autophagic adaptor protein(s) in selective autophagy of TRiC.

Under physiological conditions, cornification, which is the keratinocyte differentiation program, might be associated with autophagic degradation of TRiC. During cornification, the protein composition in cells is drastically changed, and the actin cytoskeleton is largely remodeled to be associated with adherence junctions. Importantly, all CCT subunits of TRiC have been previously shown to be increased in Atg7-deficient keratinocytes [50]. Accordingly, complete remodeling of the actin network during cornification may induce autophagic degradation of TRiC. Another putative physiological condition necessary for autophagic degradation of TRiC induction might be cell cycle arrest. When proper dynamics of actin networks are disrupted, actin signals stop cell cycle progression through unidentified mechanisms, suggesting that the correct actin network is sensed at actin checkpoints [51, 52]. Indeed, a correct actin network is required for cell cycle progression [53]. Thus, the amount of actin is tightly regulated. If the cell cycle checkpoint is activated, then TRiC continuously interacts with unfolded actin to maintain actin dynamics, and then, TRiC degradation might be induced because of the prolonged conditions conducive to cell cycle arrest.

Collectively, through analysis using TCP1α-RFP-GFP knock-in cells, we identified a novel type of autophagy, TRiCphagy, that is important for quality control of malfunctional TRiC action under the regulation of actin dynamics. Large protein complexes, including ribosomes and proteasomes, are also known to be degraded by autophagy. Ribophagy and proteaphagy were previously induced by functional inhibition using translational or proteasomal inhibitors, respectively [13, 54]. These results imply that functional inhibition of large protein complexes is a common feature in autophagy. Further investigation of degradation of TRiC by autophagy may provide new insight into the regulation of autophagy and actin homeostasis.

## Materials and methods

### Cell culture

HeLa cells (American Type Culture Collection, ATCC) were cultured in Dulbecco’s modified Eagle’s medium (Nacalai Tesque, Kyoto, Japan) supplemented with 10% fetal bovine serum (FBS; MP bio, Ringmer, UK), 50 mg/ml penicillin and streptomycin (regular medium) in a 5% CO_2_ incubator at 37 °C. Tetracycline-on (Tet-on) cells were generated by lentiviral transduction with a pCW57.1 vector (Addgene plasmid 41393, David Root lab) containing a single-vector Tet-on component and cultured in the presence of 1 µg/ml doxycycline (Clontech, Mountain View, CA, USA) during induction. For starvation treatment, cells were washed with PBS and incubated in amino acid-free DMEM without serum (starvation medium (Wako)). The cells were treated with 0.03 µM bafilomycin A_1_ (LC Laboratories, Woburn, MA, USA) to inhibit lysosomal degradation. The cells were incubated with 1 µM or 10 µM cytochalasin D (Cayman Chemical, Ann Arbor, MI, USA), 100 µM arsenite (Sigma-Aldrich, St. Louis, MO, USA), 20 µM CCCP (Nacalai Tesque), 2 µg/ml tunicamycin (Sigma), 5 µg/ml nocodazole (Cayman Chemical), 0.1 µg/ml latrunculin A (Cayman Chemical), and 1 µM cucurbitacin E (Cayman Chemical) to induce actin stress.

### Generation of HeLa TCP1α-RFP-GFP knock-in cells using CRISPR/Cas9 gene editing

The sgRNA sequence targeting exon 11 of human *TCP1 alpha* gene was designed using the CHOPCHOP tool [23, 55]. The sgRNA sequence for *TCP1 alpha* (5′- TTTTACGTTCTGGGTTA-3′) was inserted into a pSpCas9(BB)‐2A‐Puro (pX459) plasmid (Addgene plasmid 62988, Feng Zhang lab) into BspEI sites using standard techniques. To insert RFP-GFP into exon 11 of TCP1α (amino acids 476–477), a donor vector was constructed by fusing the RFP-GFP tag with upstream and downstream homology arms (700-bp each) into a pBluescript vector using Gibson assembly. HeLa cells were transfected with the donor and sgRNA expression vectors using PEI max. Six days posttransfection, the cells were trypsinized and resuspended in DMEM containing 10% FBS without phenol red. RFP- and GFP-positive cells were sorted using a Cell Sorter SH800 (Sony) and plated on a 96-well plate. The expanded single-cell colonies were screened by immunoblotting with anti-TCP1α and anti-RFP antibodies.

### Generation of a KO-cell line using CRISPR/Cas9 gene editing

SgRNA sequences for KO were designed using CHOPCHOP (FIP200:Atg5:Atg9:p62:NBR1: NDP52: Optinerurin:TAX1BP1:TOLLIP: TRIM5: NUFIP1: NCOA4 (Fig. S8)) and cloned into lentiGuide-puro (Addgene; 52963, Feng Zhang lab). HeLa TCP1α-RFP-GFP knock-in cells stably expressing FLAG-Cas9 were infected with lentivirus encoding the indicated sgRNA. Puromycin selection was started 24 h postinfection. After culture for more than 7 days, the cells were used in experiments as a KO cell line.

### Plasmids

To generate a knock-in targeting vector of TCP1α-RFP-GFP, TCP1α genomic homology arms were amplified from genomic DNA of HeLa cells using the following primers (left arm, forward, 5′- tatcgataagcttgatatcgTCACAGTGATACGAGCAGTTATACG-3′, and reverse, 5′-gagccacctccggatccAACCTGGGCCTCATTATGAAA-3′; right arm, forward, 5′-ggatcaggtggaggctccAACCCAGAACGTAAAAATCTAAAA-3′, and reverse, 5′-cggccgctctagaactagtgACAGCTTGTACTTTACTTTAATGTGTAATACTCA-3′), and inserted into a pBluescript vector with RFP/mCherry and GFP/sfGFP. Plasmids encoding WT β-actin, β-actin mutant 360A5-, A138P-, L140P-, G146D-, D157N-, D179G-, K336L- or S348L-RFP-GFP were generated by Gibson assembly using PCR products consisting of β-actin obtained from HeLa cDNAs inserted into pCW 57.1 with RFP and GFP. pCW RFP-GFP had been previously generated. pCMV-VSV-G (Addgene plasmid #8454, Bob Weinberg lab) and psPAX2 (Addgene plasmid #12260, Didier Trono lab) were used for lentivirus production.

### Antibodies

A rabbit polyclonal anti-LAMP1 antibody was a gift from Y. Tanaka (Kyushu University, Fukuoka, Japan). Mouse monoclonal anti-GFP (clone mFX75, cat. no. 012–22541), mouse monoclonal anti-β-actin (clone 2F3, cat. no. 013–24553), and mouse monoclonal anti-α-tubulin (cat. no. 071-25031) antibodies were purchased from Wako. Mouse monoclonal anti-HSP90 (610419) antibody was purchased from BD Bioscience (Tokyo, Japan). Mouse monoclonal anti-RFP (cat. no. M204-3), rabbit polyclonal anti-Atg9A (cat. no. PD042), and rabbit monoclonal anti-p62 (cat. no. PM045) antibodies were purchased from MBL. Rabbit polyclonal anti-Atg5 (cat. no. 10181-2-AP) and Rabbit polyclonal anti-RB1CC1 (cat. no.17250-1-AP) were purchased from proteintech (Illinois, USA). Rabbit monoclonal anti-TCP1α antibody was purchased from Abcam (ab92587, Cambridge, UK).

### Lentiviral infection and stable cell line generation

Stable cell lines were generated using a lentiviral expression system. HEK293FT cells were transiently cotransfected with lentiviral vectors using PEI MAX reagent (Polysciences, Warrington, PA, USA). Four hours after transfection, the medium was replaced with fresh culture medium. After culturing for 72 h, growth medium containing the lentivirus was collected. HeLa cells were incubated with collected virus-containing medium for 48 h. Uninfected cells were removed using 1 μg/ml puromycin, 5 μg/ml blasticidin S (Wako), or 100 µg/ml hygromycin (Wako).

### Flow cytometry

The cells detached with trypsin-EDTA were resuspended in 5% newborn calf serum (NBS) and 1 µg/ml DAPI in PBS, passed through a 70 µm cell strainer, and analyzed by a CytoFLEX S flow cytometer equipped with NUV 375 nm (DAPI), 488 nm (GFP), and 561-nm (RFP) lasers (Beckman Coulter). Dead cells were detected by DAPI staining. In each sample, 10,000 cells were obtained, and the R/G fluorescence ratios, the red fluorescence intensity divided by the green fluorescence intensity, were calculated for RFP-positive cells.

### Fluorescence microscopy

Cells expressing fluorescent‐tagged protein were grown on coverslips and fixed in 3.7% formaldehyde in PBS for 15 min. For immunostaining, fixed cells were permeabilized with 50 µg/ml digitonin in PBS for 5 min, blocked with 10% NBS in PBS for 30 min, and incubated with primary antibodies for 1 h. After washing, the cells were incubated with Alexa Fluor 647–conjugated goat anti-rabbit IgG secondary antibodies (Thermo Fisher Scientific) for 1 h. The stained cells were observed under a confocal laser microscope (FV1000 IX81; Olympus) using a 100× oil immersion objective lens with an NA of 1.40. The images were acquired using FV10-ASW 2.1 imaging software.

### Immunoblotting

Cells were washed with cold PBS and lysed in lysis buffer (1% Triton X-100; 50 mM Tris/HCl (pH 7.5); 1 mM EDTA; and 150 mM NaCl) supplemented with protease-inhibitor cocktail (EDTA-free) (Nacalai Tesque) and 1 mM phenylmethanesulfonyl fluoride for 15 min at 4 °C. The lysates were clarified by centrifugation at 20,630 × *g* for 5 min to remove the nuclei. For SDS/polyacrylamide gel electrophoresis (SDS/PAGE), the samples were mixed into SDS sample buffer (83 mM Tris (pH 6.8), 17 mg/ml SDS, 15.5 mg/ml DTT, 5% glycerol, and 0.04% bromophenol blue)) and boiled at 95 °C for 5 min. For native/polyacrylamide gel electrophoresis (native/PAGE) performed as part of the actin folding assays, the sample were mixed into native sample buffer(16% glycerol, 16 mM MOPS-KOH(pH 7.2), and 0.04% bromophenol blue) [56]. Twenty micrograms of protein was added to each lane in the gel and separated by SDS-PAGE or native PAGE and transferred to a polyvinylidene difluoride membrane (Millipore, Billerica, MA, USA). Immunoblot analysis was performed with the indicated antibodies, and the immunoreactive proteins were visualized using ImmunoStar Zeta (Wako).

### Sucrose density gradient

Gradients (2.0 ml) were prepared in a 7 × 20 mm centrifuge tube by successively layering with 0.4 ml of 25, 20, 15, 10, and 5% sucrose (W/V) in PBS. Lysates (postnuclear supernatant (PNS)) were prepared as described above. PNS (0.2 ml) were loaded on top of the gradients and centrifuged for 2 h at 55,000 r.p.m. at 4 °C (CS 150FNX, rotor: S55S, Hitachi, Tokyo, Japan). After centrifugation, eleven 0.2 ml fractions were collected from the top. Each fraction was analyzed by immunoblotting.

### Immunoprecipitation

Cell lysates were prepared with lysis buffer as described above. After centrifugation at 20,630 × *g* for 30 min at 4 °C, the supernatant was incubated with or without 0.01 µg/µl antibodies for 1 h and was added to protein G Sepharose (GE healthcare, Uppsala, Sweden) or GFP traps (typically with a 10 µl-resin volume) and then rotated for 3 h. The beads were washed 4 times in lysis buffer and boiled in sample buffer. The samples were subsequently separated by SDS-PAGE and analyzed by immunoblotting,

### MTT assay

HeLa WT and TCP1α-RG cells were measured by 3-(4,5-dimethylthiazol-2-yl)-2,5-diphenyltetrazolium bromide (MTT) assay (Nacalai Tesque). The cells were seeded into 96-well plates at a density of 1.5 × 10^3^ cells per well, and then the proliferation rate was measured by MTT assays reagent after 1, 2, 3, and 4 days culture.

## Supplementary information

Sup figures

## Data Availability

The data in this work are available with the approval of corresponding authors in reasonable requirement.
