# Supplementary material for: Disruption of actin dynamics induces autophagy of the eukaryotic chaperonin TRiC/CCT
Source: Cell Death Discov. 2022 Jan 25;8:37. doi: 10.1038/s41420-022-00828-6 (PMC8789831; doi:10.1038/s41420-022-00828-6)
Supplement: Supplementary file 1 — Sup figures [file 41420_2022_828_MOESM1_ESM.pdf]

A

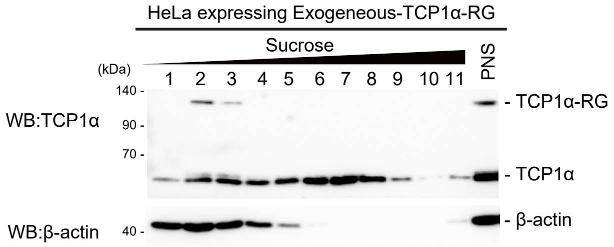

B

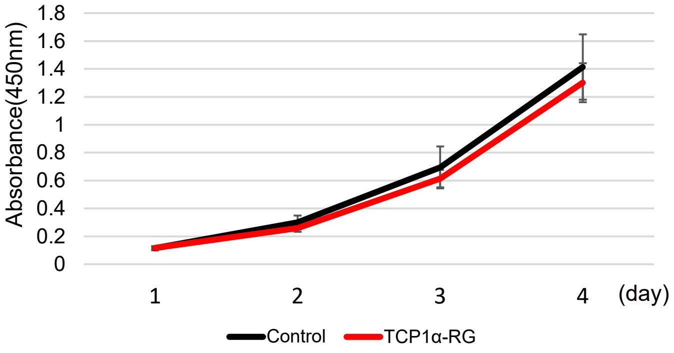

Figure S1 TCP1α-RG cells charactarizations. (Figure1 related). (A) HeLa cells stably expressing exogenous TCP1α-RFP-GFP (TCP1α-RG) were generated by a lentivirus system and homogenized. The resulting post-nuclear supernatants (PNSs) were fractionated with sucrose density gradients and analyzed by immunoblotting using antibodies against TCP1α and β-actin. (**B)** Proliferation rate of HeLa WT cells and TCP1α-RG cells were measured by MTT assay after 1,2,3 and 4 days culture. The graph shows absorbance (450nm) (measured value - blank value). The data are presented as the means ± SEM (n=3). Knock-in of TCP1α-RG did not affect the Proliferation rate.

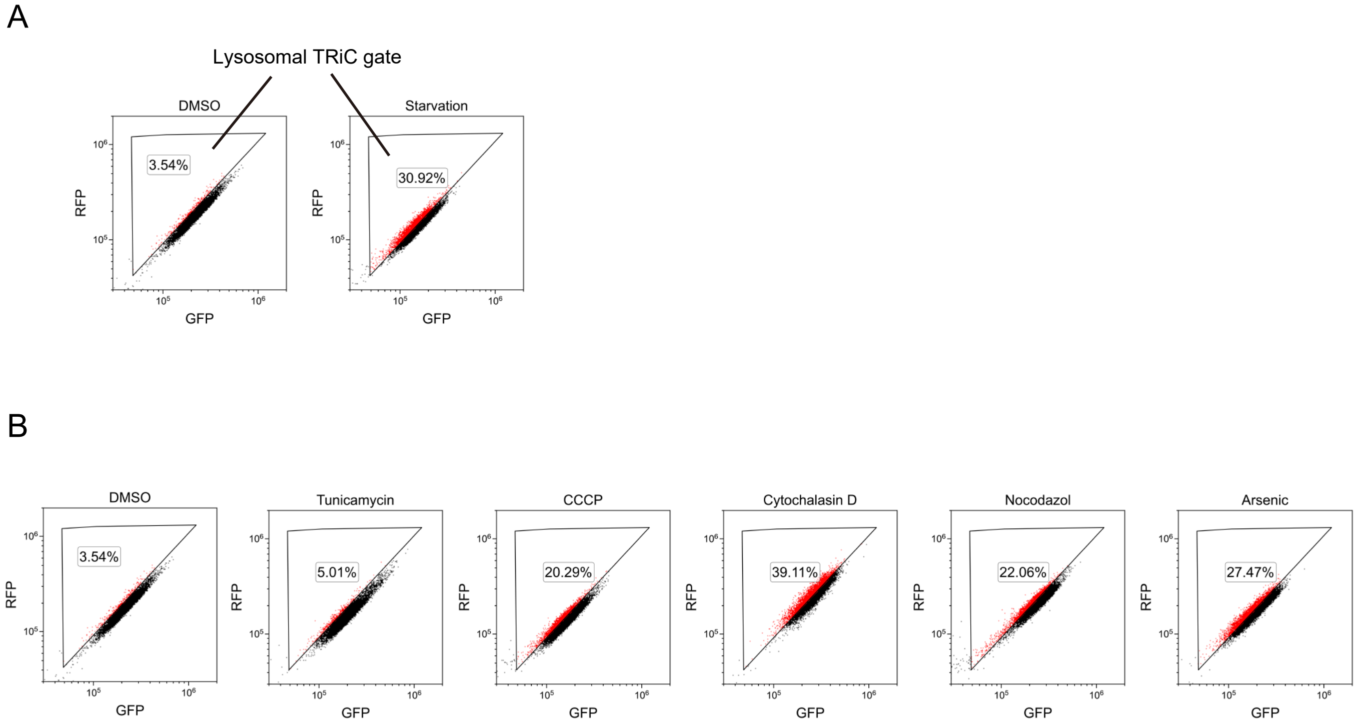

Figure S2 Flow cytometry analysis of HeLa TCP1α-RFP-GFP cells using a lysosomal TRiC gate (related to Figure 2). (**A, B**) HeLa TCP1α-RFP-GFP cells were treated with DMSO, starvation, tunicamycin, CCCP, cytochalasin D, nocodazole or arsenic for 24 h and analyzed by flow cytometry. The "Lysosomal TRiC gate" showed a decrease in GFP signals but not RFP fluorescence compared with these levels after DMSO treatment. Dot plots show the lysosomal TRiC gate-positive cell population (%).

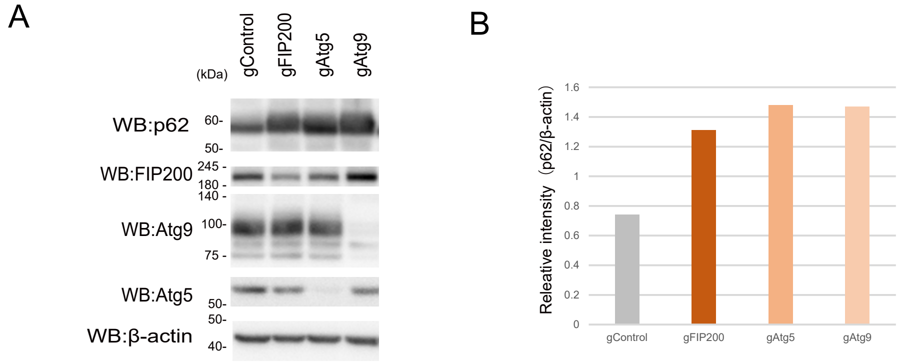

Figure S3 Analysis of autophagy knock-out cells generated by CRISPR (related to Figure 3A-C) (A) HeLa TCP1α-RFP-GFP knock-in cells stably expressing FLAG-Cas9 were infected with lentivirus encoding the indicated sgRNA(gControl (nontargeting gRNA for the human genome), gFIP200, gAtg5, or gAtg9). These cells were analyzed by immunoblotting using antibodies against FIP200, Atg5, Atg9, p62, β-actin. (B) Releative level of p62. The bar graph shows p62/β-actin ratio of intensity. All autophagy KO cells accumulated p62, which is autophagy substrate.

A

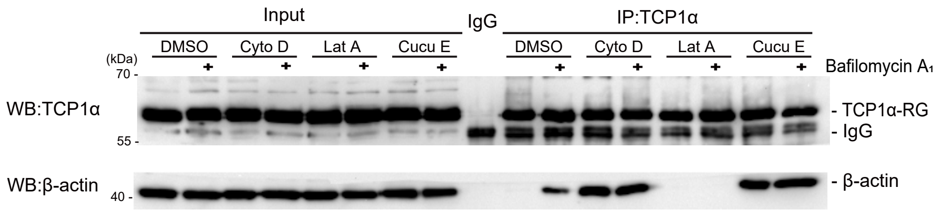

B

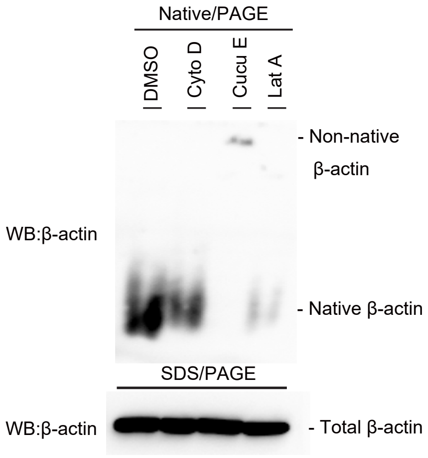

Figure S4 Actin (de)polymerization inhibitors inactivate TRiC. (**A**) WT HeLa cells were treated with DMSO, cytochalasin D (Cyto D), latrunculin A (Lat A) or cucurbitacin E (Cucu E)with or without bafilomycin A_1 (baf) for 24 h, lysed in lysis buffer and subjected to immunoprecipitation using an anti-TCP1α antibody. The purified samples were analyzed by immunoblotting using antibodies against TCP1α and β-actin. (**B**) WT HeLa cells were treated with DMSO, cytochalasin D (Cyto D), cucurbitacin E (Cucu E), or latrunculin A (Lat A)and analyzed by native PAGE and immunoblotting using an antibody against β-actin. Bottom panel: The samples boiled in SDS buffer were analyzed by immunoblotting using antibodies against β-actin.

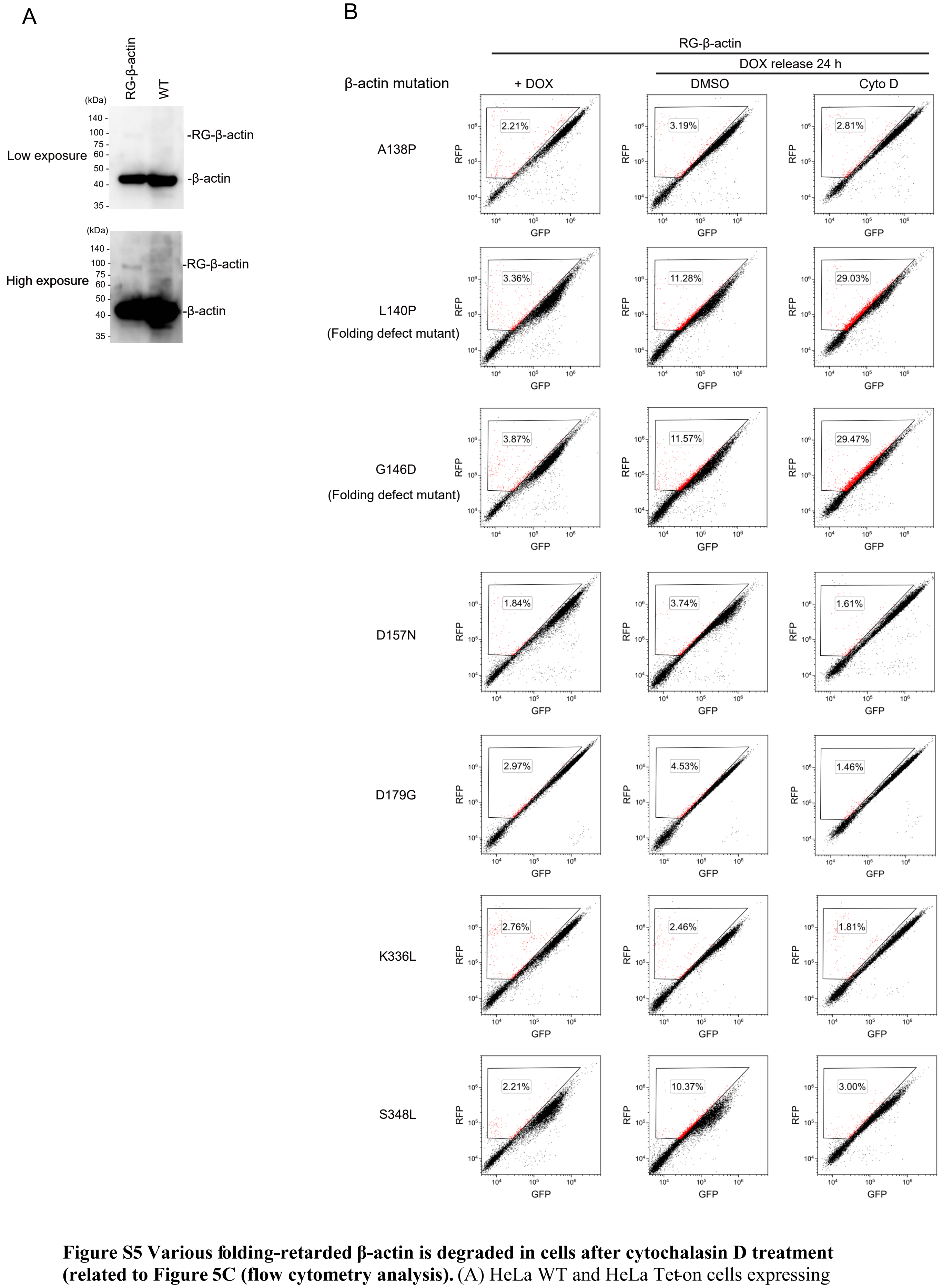

Figure S5 Various folding-retarded β-actin is degraded in cells after cytochalasin D treatment (related to Figure 5C (flow cytometry analysis). (A) HeLa WT and HeLa Tet-on cells expressing RFP-GFP(RG)-β-actin were cultured in the presence of Doxycycline (Dox) and analyzed by immunoblotting using antibodies against β-actin. (B) HeLa Tet-on cells expressing RFP-GFP(RG)-β-actin, A138P, L140P, G146D, D157N, D179G, K336L or S348L were cultured in the presence of doxycycline (Dox). After removal of Dox, the cells were treated with DMSO or cytochalasin D (Cyto D) for 24 h and analyzed by flow cytometry. Dot plots show the lysosomal TRiC gate-positive cell population (%).

A

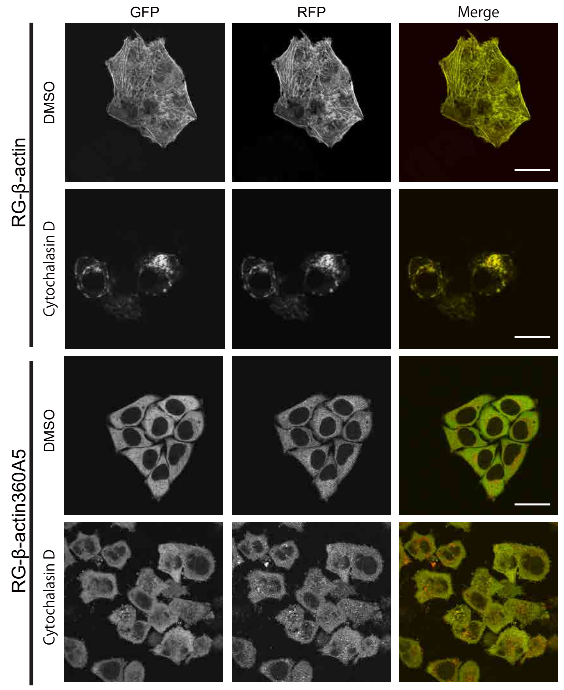

B

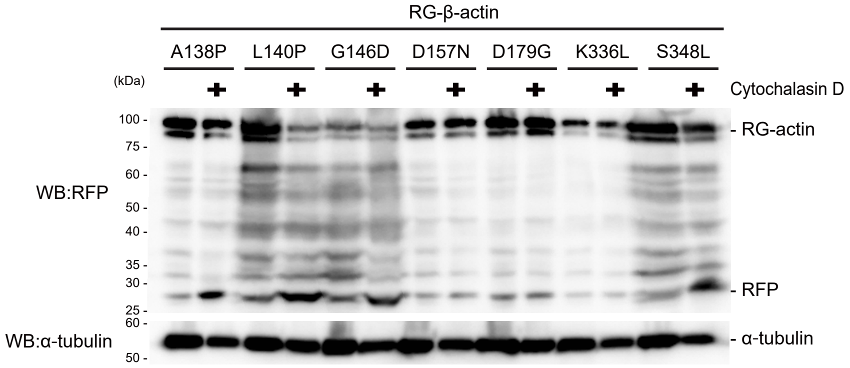

Figure S6 Various folding-retarded β-actin is degraded in cells after cytochalasin D treatment (immunoblotting and fluorescence microscopic analysis).
(A) HeLa cells stably expressing RFP-GFP(RG)-β-actin or RG-β-actin 360A5 were treated with DMSO or cytochalasin D (Cyto D) for 24 h and then fixed and analyzed by confocal microscopy. Scale bar, 20 μm. (B) HeLa Tet-on cells expressing RFP-GFP(RG)-β-actinA138P, L140P, G146D, D157N, D179G, K336L or S348L were cultured in the presence of doxycycline (Dox), treated with DMSO or cytochalasin D (Cyto D) for 24 h and analyzed by immunoblotting using antibodies against RFP and α-tubulin.

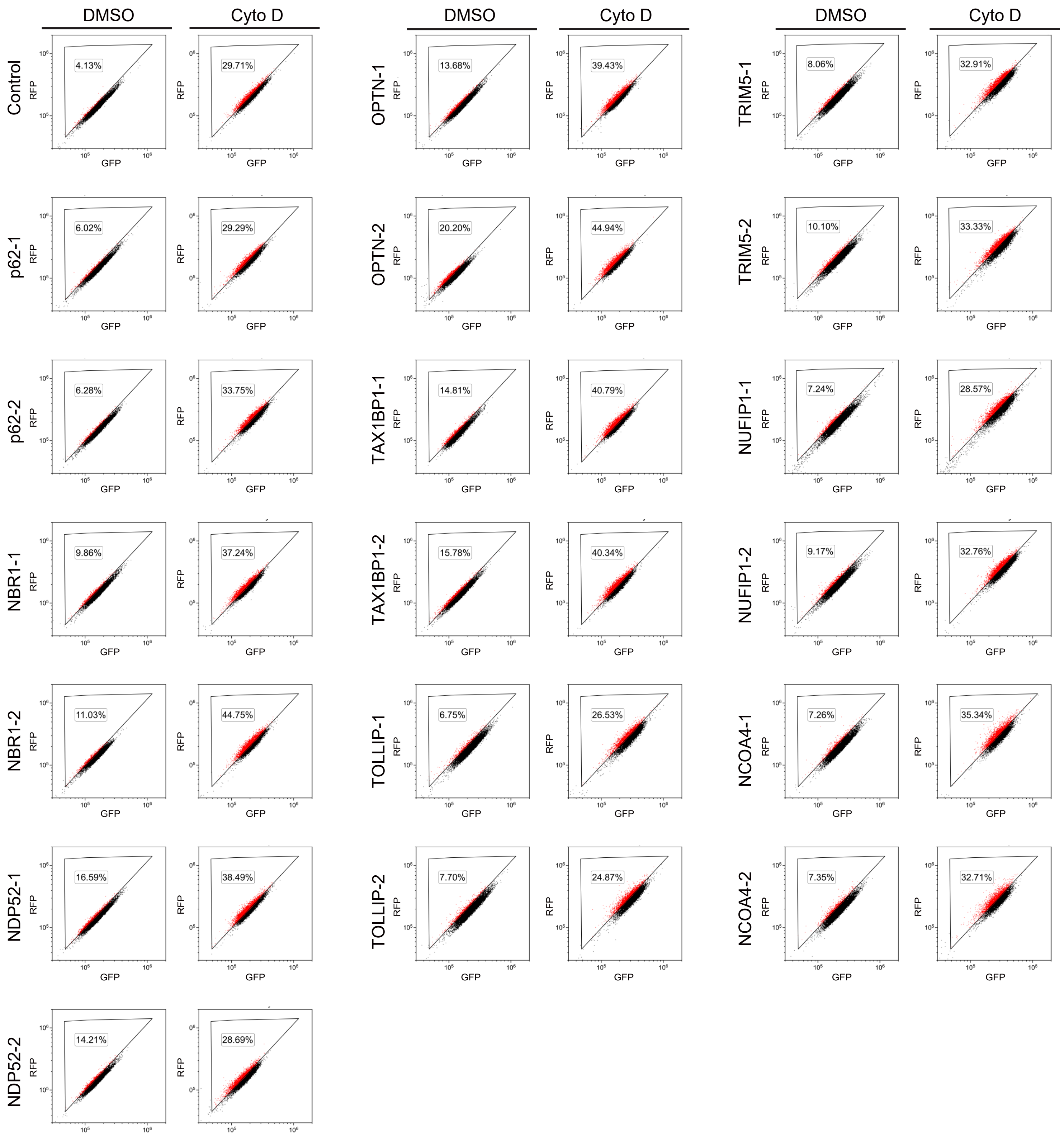

Figure S7 Flow cytometry analysis of autophagy adaptor KO cells.
HeLa TCP1α-RFP-GFP knock-in cells stably expressing FLAG-Cas9 were infected with lentivirus encoding the indicated sgRNA (gControl (nontargeting gRNA for the human genome), gp62, gNBR1, gNDP52, gOPTN, gTAX1BP1, gTOLLIP,gTRIM5, gNUFIP1, gNCOA4). These cells were treated with DMSO or Cytochalasin D for 24 h and analyzed by flow cytometry. Dot plots show the lysosomal TRiC gate-positive cell population (%).

	sgRNA sequences
RB1CC1 (FIP200)	GGCTGCAATCATGGCCAACC
Atg9	AGGATATTCGAGAGAAGAAG
Atg5	AAGAGTAAGTTATTTGACGT
p62-1	AATGGCCATGTCCTACGTGA
p62-2	AGGGCTTCTCGCACAGCCGC
NBR1-1	CTGATCCAGAAAATACAACT
NBR1-2	TACTATTCAAATAAAATACC
NDP52-1	TGAGTATTACACCTTCATGT
NDP52-2	GTATTACCAGTTCTGCTATG
OPTN-1	TCCCAGGGCCGAAGCGGAGC
OPTN-2	GTTCAGACACGATGCCCAAC
TAX1BP1-1	AATATACCAACCCAATCTTT
TAX1BP1-2	TCTGAAACTCCGTCTTCAGA
TOLLIP-1	GTACATCGGTGAGCTCCCGC
TOLLIP-2	AATGCGGTCGTCCATGGAGA
TRIM5-1	CTGAGAACATACGGCCTAAT
TRIM5-2	CTCAAAATCTGCCAAGACGT
NUFIP1-1	GGAGACGCATGCCACCCGAT
NUFIP1-2	TTCTAGAAACTATCCAACTC
NCOA4-1	GTGCATCACTACACCTCAAA
NCOA4-2	GCATGAGCCATCAAGTGCTC

Figure S8 SgRNA sequences used in this study.
